# Retraction: How Traumatic Violence Permanently Changes Shopping Behavior

**DOI:** 10.3389/fpsyg.2017.02140

**Published:** 2017-11-27

**Authors:** Frontiers Editorial Office

The journal retracts the 6 September 2016 article cited above.

Following publication, concerns were brought to the attention of the publisher regarding the validity of the article's findings. Adhering to our complaints procedure, Frontiers engaged an expert to assess the raw data for the study. The conclusion from this assessment, supported by the Specialty and Field Chief Editor, is that there is no empirical support for the conclusions of the article.

